# {*N*′,*N*′′-Bis[2,6-bis­(1-methyl­ethyl)phen­yl]-*N*,*N*-dimethyl­guanidinato-κ^2^ *N*′,*N*′′}dibromido­borane

**DOI:** 10.1107/S1600536810004988

**Published:** 2010-02-13

**Authors:** Holger Braunschweig, Rian D. Dewhurst, Katrin Schwab, Katharina Wagner

**Affiliations:** a Institut für Anorganische Chemie, Universität Würzburg, Am Hubland, D-97074 Würzburg, Germany

## Abstract

In the mol­ecular structure of the title compound, C_27_H_40_N_3_BBr_2_, the B atom is connected to two bromide substituents and a guanidinate scaffold, forming a four–membered ring. An aryl group is connected to each N atom in the ring that contains two isopropyl groups in positions 2 and 6.

## Related literature

For the synthesis of a similar compound, see: Findlater *et al.* (2006 ▶).
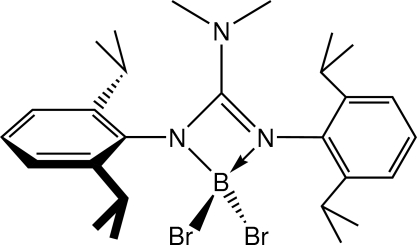


## Experimental

### 

#### Crystal data

C_27_H_40_BBr_2_N_3_
*M* *_r_* = 577.23
Monoclinic, 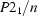

*a* = 9.996 (3) Å
*b* = 16.080 (5) Å
*c* = 18.464 (6) Å
β = 93.505 (5)°
*V* = 2962.3 (16) Å^3^
*Z* = 4
Mo *K*α radiation
μ = 2.76 mm^−1^
*T* = 166 K
0.95 × 0.34 × 0.18 mm

#### Data collection

Bruker SMART APEX CCD area-detector diffractometer
Absorption correction: multi-scan (*SADABS*; Bruker, 2008 ▶) *T*_min_ = 0.260, *T*_max_ = 0.609
79220 measured reflections
7424 independent reflections
6550 reflections with *I* > 2σ(*I*)
*R*_int_ = 0.030

#### Refinement

*R*[*F* ^2^ > 2σ(*F* ^2^)] = 0.026
*wR*(*F* ^2^) = 0.069
*S* = 1.04
7424 reflections
308 parameters
H-atom parameters constrained
Δρ_max_ = 0.63 e Å^−3^
Δρ_min_ = −0.52 e Å^−3^

### 

Data collection: *SMART* (Bruker, 1997 ▶); cell refinement: *SAINT* (Bruker, 1997 ▶); data reduction: *SAINT*; program(s) used to solve structure: *SHELXS97* (Sheldrick, 2008 ▶); program(s) used to refine structure: *SHELXL97* (Sheldrick, 2008 ▶); molecular graphics: *ORTEP-3* (Farrugia, 1997 ▶); software used to prepare material for publication: *SHELXTL* (Sheldrick, 2008 ▶).

## Supplementary Material

Crystal structure: contains datablocks I, global. DOI: 10.1107/S1600536810004988/rk2190sup1.cif

Structure factors: contains datablocks I. DOI: 10.1107/S1600536810004988/rk2190Isup2.hkl

Additional supplementary materials:  crystallographic information; 3D view; checkCIF report

## Figures and Tables

**Fig. 1.**
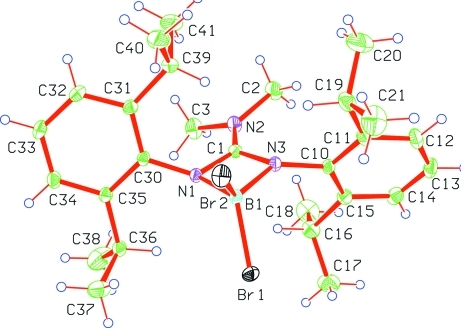
The molecular structure of title compound. Displacement ellipsoids are drawn at the 30% probability level. The H atoms are presented as a small spheres of arbitrary radius.

## supplementary crystallographic information

### Comment

The title compound is a new congener of a boron amidinate and was prepared by an insertion reaction of bis(2,6-diisopropylphenyl)carbodiimide into the B—N bond of 1,1-dibromo-*N*,*N*-dimethylboramine.

Findlater *et al.* (2006) reported the first example of the insertion of a carbodiimide into a boron–nitrogen bond to generate [(*Me*_3_Si)_2_NC{N*Cy*}_2_]BCl_2_ that has a boron–halide bond. Before this, their preparation was only known *via* salt metathesis reactions of the appropriate lithium amidinates with haloboranes.

As shown in Fig. 1, the B—Br distances and the B—N distances are comparable with those found in [(*Me*_3_Si)_2_NC{N*Cy*}_2_]BCl_2_ (Findlater *et al.*, 2006). The angles Br1—B1—Br2 and N1—B1—N3 are smaller than the corresponding angles in [(*Me*_3_Si)_2_NC{N*Cy*}_2_]BCl_2_ (Findlater *et al.*, 2006). This could be due to the higher steric hindrance of the substituted aryl group than the cyclohexyl group in the reference substance.

### Experimental

To prepare the title compound, bis(2,6-diiso-propylphenyl)carbodiimide (0.50 g, 1.38 mmol) dissolved in 2 ml toluene, and 1,1-dibromo-*N*,*N*-dimethylaminoborane (0.23 g, 1.08 mmol) dissolved in 2 ml toluene were combined and stirred for 20 h at ambient temperature. After a few minutes a precipitate was noticed. All volatiles were removed under reduced pressure. Recrystallization at ambient temperature from benzene gave colourless crystals of title compound (0.51 g, 0.88 mmol, yield 81%).

### Refinement

The H atoms were placed at idealized positions and treated as riding atoms with C—H = 0.96 Å (CH_3_), 0.98 Å (aliphatic CH) and 0.93 Å (aromatic CH). *U*_iso_(H) values were fixed at 1.5 times (for primary H atoms) and 1.2 times (tertiary or aromatic H atoms) *U*_eq_ of the attached C atoms.

### Crystal data

**Table:**

C_27_H_40_BBr_2_N_3_	*F*(000) = 1192
*M**_r_* = 577.23	*D*_x_ = 1.294 Mg m^−^^3^
Monoclinic, *P*2_1_/*n*	Mo *K*α radiation, λ = 0.71073 Å
Hall symbol: -P 2yn	Cell parameters from 8028 reflections
*a* = 9.996 (3) Å	θ = 2.3–28.3°
*b* = 16.080 (5) Å	µ = 2.76 mm^−^^1^
*c* = 18.464 (6) Å	*T* = 166 K
β = 93.505 (5)°	Plate, colourless
*V* = 2962.3 (16) Å^3^	0.95 × 0.34 × 0.18 mm
*Z* = 4	

### Data collection

**Table:**

Bruker SMART APEX CCD area-detector diffractometer	7424 independent reflections
Radiation source: sealed tube	6550 reflections with *I* > 2σ(*I*)
graphite	*R*_int_ = 0.030
φ and ω scans	θ_max_ = 28.4°, θ_min_ = 1.7°
Absorption correction: multi-scan (*SADABS*; Bruker, 2008)	*h* = −13→13
*T*_min_ = 0.260, *T*_max_ = 0.609	*k* = −21→21
79220 measured reflections	*l* = −24→24

### Refinement

**Table:**

Refinement on *F*^2^	Primary atom site location: structure-invariant direct methods
Least-squares matrix: full	Secondary atom site location: difference Fourier map
*R*[*F*^2^ > 2σ(*F*^2^)] = 0.026	Hydrogen site location: inferred from neighbouring sites
*wR*(*F*^2^) = 0.069	H-atom parameters constrained
*S* = 1.04	*w* = 1/[σ^2^(*F*_o_^2^) + (0.0374*P*)^2^ + 1.1023*P*] where *P* = (*F*_o_^2^ + 2*F*_c_^2^)/3
7424 reflections	(Δ/σ)_max_ = 0.001
308 parameters	Δρ_max_ = 0.63 e Å^−^^3^
0 restraints	Δρ_min_ = −0.52 e Å^−^^3^

### Special details

**Table:**

Geometry. All s.u.'s (except the s.u. in the dihedral angle between two l.s. planes) are estimated using the full covariance matrix. The cell s.u.'s are taken into account individually in the estimation of s.u.'s in distances, angles and torsion angles; correlations between s.u.'s in cell parameters are only used when they are defined by crystal symmetry. An approximate (isotropic) treatment of cell s.u.'s is used for estimating s.u.'s involving l.s. planes.
Refinement. Refinement of *F*^2^ against ALL reflections. The weighted *R*–factor wR and goodness of fit *S* are based on *F*^2^, conventional *R*–factors *R* are based on *F*, with *F* set to zero for negative *F*^2^. The threshold expression of\ *F*^2^ > σ(*F*^2^) is used only for calculating *R*–factors(gt) etc. and is not relevant to the choice of reflections for refinement. *R*–factors based on *F*^2^ are statistically about twice as large as those based on *F*, and *R*–factors based on ALL data will be even larger.

### Fractional atomic coordinates and isotropic or equivalent isotropic displacement parameters (Å^2^)

**Table:**

	*x*	*y*	*z*	*U*_iso_*/*U*_eq_	
B1	0.01926 (15)	0.04337 (9)	0.24662 (9)	0.0231 (3)	
Br1	−0.131845 (15)	0.011137 (10)	0.306711 (9)	0.03424 (5)	
Br2	0.046698 (17)	−0.047479 (10)	0.174634 (9)	0.03696 (5)	
C30	0.27818 (13)	0.03635 (8)	0.30412 (8)	0.0223 (3)	
C31	0.37833 (14)	0.03839 (9)	0.25355 (8)	0.0264 (3)	
C32	0.50122 (15)	0.00155 (10)	0.27394 (9)	0.0323 (3)	
H32A	0.5698	0.0034	0.2421	0.039*	
C33	0.52377 (15)	−0.03759 (10)	0.34008 (9)	0.0336 (3)	
H33A	0.6065	−0.0619	0.3523	0.040*	
C35	0.29912 (14)	−0.00356 (9)	0.37141 (8)	0.0244 (3)	
C36	0.19185 (16)	−0.00687 (10)	0.42605 (8)	0.0313 (3)	
H36A	0.1150	0.0257	0.4064	0.038*	
C39	0.36010 (16)	0.07683 (11)	0.17835 (9)	0.0339 (3)	
H39A	0.2670	0.0956	0.1711	0.041*	
C1	0.12374 (14)	0.15035 (8)	0.26028 (7)	0.0229 (3)	
C3	0.28701 (19)	0.22944 (11)	0.33429 (10)	0.0426 (4)	
H3A	0.2769	0.1838	0.3671	0.064*	
H3B	0.2707	0.2808	0.3587	0.064*	
H3C	0.3764	0.2295	0.3181	0.064*	
C2	0.17392 (19)	0.29175 (10)	0.22274 (11)	0.0417 (4)	
H2A	0.1220	0.2753	0.1797	0.063*	
H2B	0.2602	0.3110	0.2099	0.063*	
H2C	0.1284	0.3356	0.2465	0.063*	
C10	−0.09603 (14)	0.19049 (8)	0.19551 (8)	0.0240 (3)	
C11	−0.12695 (15)	0.19503 (9)	0.12043 (8)	0.0297 (3)	
C12	−0.22852 (18)	0.24924 (11)	0.09581 (10)	0.0383 (4)	
H12A	−0.2511	0.2530	0.0463	0.046*	
C19	−0.05507 (19)	0.14327 (12)	0.06641 (9)	0.0406 (4)	
H19A	0.0129	0.1099	0.0936	0.049*	
C20	0.0170 (3)	0.19737 (19)	0.01262 (13)	0.0737 (7)	
H20A	0.0814	0.2322	0.0387	0.111*	
H20B	−0.0473	0.2315	−0.0143	0.111*	
H20C	0.0620	0.1623	−0.0202	0.111*	
C21	−0.1521 (3)	0.08357 (17)	0.02629 (13)	0.0664 (7)	
H21A	−0.1924	0.0481	0.0607	0.100*	
H21B	−0.1044	0.0503	−0.0067	0.100*	
H21C	−0.2207	0.1147	−0.0003	0.100*	
C15	−0.16645 (15)	0.23739 (9)	0.24479 (8)	0.0287 (3)	
C16	−0.14292 (19)	0.23165 (11)	0.32672 (9)	0.0383 (4)	
H16A	−0.0768	0.1877	0.3377	0.046*	
C18	−0.0876 (3)	0.31294 (14)	0.35935 (12)	0.0603 (6)	
H18A	−0.0052	0.3266	0.3380	0.090*	
H18B	−0.0714	0.3065	0.4108	0.090*	
H18C	−0.1515	0.3567	0.3497	0.090*	
C17	−0.2733 (2)	0.20788 (15)	0.36188 (12)	0.0553 (5)	
H17A	−0.3088	0.1576	0.3402	0.083*	
H17B	−0.3376	0.2519	0.3544	0.083*	
H17C	−0.2549	0.1993	0.4130	0.083*	
N1	0.14975 (11)	0.07362 (7)	0.28819 (6)	0.0218 (2)	
N2	0.19091 (13)	0.22050 (8)	0.27179 (7)	0.0300 (3)	
N3	0.00984 (11)	0.13479 (7)	0.21945 (6)	0.0226 (2)	
C13	−0.29584 (17)	0.29719 (10)	0.14300 (11)	0.0399 (4)	
H13A	−0.3623	0.3337	0.1253	0.048*	
C14	−0.26533 (17)	0.29146 (10)	0.21643 (10)	0.0367 (4)	
H14A	−0.3117	0.3244	0.2479	0.044*	
C40	0.3863 (2)	0.01302 (14)	0.11895 (10)	0.0483 (5)	
H40A	0.3637	0.0370	0.0722	0.072*	
H40B	0.3324	−0.0355	0.1254	0.072*	
H40C	0.4793	−0.0023	0.1222	0.072*	
C41	0.4512 (2)	0.15212 (14)	0.17041 (12)	0.0533 (5)	
H41A	0.4335	0.1925	0.2069	0.080*	
H41B	0.4340	0.1763	0.1232	0.080*	
H41C	0.5432	0.1349	0.1762	0.080*	
C37	0.1445 (2)	−0.09584 (15)	0.43660 (14)	0.0613 (6)	
H37A	0.1082	−0.1175	0.3911	0.092*	
H37B	0.0765	−0.0965	0.4712	0.092*	
H37C	0.2188	−0.1296	0.4542	0.092*	
C38	0.2403 (3)	0.0318 (2)	0.49769 (12)	0.0706 (7)	
H38A	0.2704	0.0875	0.4896	0.106*	
H38B	0.3131	−0.0005	0.5192	0.106*	
H38C	0.1682	0.0329	0.5297	0.106*	
C34	0.42350 (15)	−0.04071 (10)	0.38811 (9)	0.0308 (3)	
H34A	0.4389	−0.0679	0.4323	0.037*	

### Atomic displacement parameters (Å^2^)

**Table:**

	*U*^11^	*U*^22^	*U*^33^	*U*^12^	*U*^13^	*U*^23^
B1	0.0203 (7)	0.0207 (7)	0.0278 (7)	−0.0006 (5)	−0.0025 (6)	0.0013 (6)
Br1	0.02314 (8)	0.03269 (9)	0.04722 (10)	−0.00460 (6)	0.00494 (6)	0.00839 (6)
Br2	0.04139 (10)	0.02723 (8)	0.04154 (10)	0.00203 (6)	−0.00325 (7)	−0.00983 (6)
C30	0.0186 (6)	0.0207 (6)	0.0270 (6)	−0.0008 (5)	−0.0026 (5)	−0.0007 (5)
C31	0.0221 (6)	0.0269 (7)	0.0300 (7)	−0.0030 (5)	0.0010 (5)	0.0006 (5)
C32	0.0208 (7)	0.0386 (8)	0.0378 (8)	−0.0010 (6)	0.0042 (6)	−0.0018 (6)
C33	0.0208 (7)	0.0371 (8)	0.0420 (9)	0.0045 (6)	−0.0049 (6)	−0.0013 (7)
C35	0.0215 (6)	0.0256 (7)	0.0257 (7)	−0.0002 (5)	−0.0021 (5)	−0.0010 (5)
C36	0.0264 (7)	0.0422 (9)	0.0251 (7)	0.0055 (6)	0.0002 (6)	0.0038 (6)
C39	0.0294 (7)	0.0407 (9)	0.0320 (8)	−0.0002 (6)	0.0060 (6)	0.0074 (7)
C1	0.0228 (6)	0.0219 (6)	0.0239 (6)	0.0001 (5)	0.0015 (5)	0.0010 (5)
C3	0.0425 (10)	0.0323 (8)	0.0507 (10)	−0.0078 (7)	−0.0158 (8)	−0.0042 (7)
C2	0.0437 (9)	0.0254 (8)	0.0552 (11)	−0.0059 (7)	−0.0033 (8)	0.0114 (7)
C10	0.0222 (6)	0.0208 (6)	0.0286 (7)	0.0012 (5)	−0.0007 (5)	0.0036 (5)
C11	0.0305 (7)	0.0270 (7)	0.0309 (7)	0.0000 (6)	−0.0035 (6)	0.0049 (6)
C12	0.0401 (9)	0.0332 (8)	0.0395 (9)	0.0009 (7)	−0.0131 (7)	0.0095 (7)
C19	0.0483 (10)	0.0480 (10)	0.0251 (8)	0.0086 (8)	−0.0001 (7)	0.0026 (7)
C20	0.0832 (18)	0.0905 (19)	0.0501 (13)	0.0046 (15)	0.0264 (12)	0.0144 (13)
C21	0.0759 (16)	0.0676 (15)	0.0542 (13)	0.0070 (13)	−0.0092 (11)	−0.0247 (12)
C15	0.0294 (7)	0.0225 (7)	0.0343 (8)	−0.0006 (5)	0.0031 (6)	0.0010 (6)
C16	0.0474 (10)	0.0350 (8)	0.0331 (8)	0.0056 (7)	0.0069 (7)	−0.0041 (7)
C18	0.0810 (16)	0.0488 (12)	0.0508 (12)	−0.0019 (11)	0.0009 (11)	−0.0181 (10)
C17	0.0622 (13)	0.0570 (12)	0.0496 (11)	0.0110 (10)	0.0267 (10)	0.0030 (9)
N1	0.0192 (5)	0.0200 (5)	0.0256 (5)	−0.0007 (4)	−0.0018 (4)	0.0023 (4)
N2	0.0299 (6)	0.0221 (6)	0.0371 (7)	−0.0042 (5)	−0.0061 (5)	0.0024 (5)
N3	0.0208 (5)	0.0205 (5)	0.0260 (6)	0.0005 (4)	−0.0018 (4)	0.0017 (4)
C13	0.0282 (8)	0.0291 (8)	0.0611 (11)	0.0043 (6)	−0.0082 (7)	0.0092 (7)
C14	0.0311 (8)	0.0252 (7)	0.0543 (10)	0.0054 (6)	0.0067 (7)	0.0013 (7)
C40	0.0492 (11)	0.0629 (13)	0.0332 (9)	0.0031 (9)	0.0079 (8)	0.0003 (8)
C41	0.0494 (11)	0.0510 (11)	0.0609 (12)	−0.0096 (9)	0.0156 (9)	0.0147 (10)
C37	0.0489 (12)	0.0591 (13)	0.0782 (16)	−0.0029 (10)	0.0230 (11)	0.0171 (12)
C38	0.0609 (14)	0.112 (2)	0.0396 (11)	−0.0030 (14)	0.0080 (10)	−0.0265 (13)
C34	0.0262 (7)	0.0340 (8)	0.0313 (7)	0.0026 (6)	−0.0060 (6)	0.0035 (6)

### Geometric parameters (Å, °)

**Table:**

B1—N1	1.5507 (19)	C12—H12A	0.9300
B1—N3	1.5543 (19)	C19—C21	1.524 (3)
B1—Br1	1.9966 (17)	C19—C20	1.533 (3)
B1—Br2	2.0050 (16)	C19—H19A	0.9800
C30—C35	1.403 (2)	C20—H20A	0.9600
C30—C31	1.411 (2)	C20—H20B	0.9600
C30—N1	1.4310 (17)	C20—H20C	0.9600
C31—C32	1.395 (2)	C21—H21A	0.9600
C31—C39	1.520 (2)	C21—H21B	0.9600
C32—C33	1.380 (2)	C21—H21C	0.9600
C32—H32A	0.9300	C15—C14	1.394 (2)
C33—C34	1.379 (2)	C15—C16	1.519 (2)
C33—H33A	0.9300	C16—C18	1.529 (3)
C35—C34	1.397 (2)	C16—C17	1.540 (3)
C35—C36	1.517 (2)	C16—H16A	0.9800
C36—C38	1.514 (3)	C18—H18A	0.9600
C36—C37	1.523 (3)	C18—H18B	0.9600
C36—H36A	0.9800	C18—H18C	0.9600
C39—C41	1.527 (3)	C17—H17A	0.9600
C39—C40	1.536 (3)	C17—H17B	0.9600
C39—H39A	0.9800	C17—H17C	0.9600
C1—N2	1.3231 (18)	C13—C14	1.374 (3)
C1—N3	1.3500 (18)	C13—H13A	0.9300
C1—N1	1.3563 (18)	C14—H14A	0.9300
C3—N2	1.463 (2)	C40—H40A	0.9600
C3—H3A	0.9600	C40—H40B	0.9600
C3—H3B	0.9600	C40—H40C	0.9600
C3—H3C	0.9600	C41—H41A	0.9600
C2—N2	1.464 (2)	C41—H41B	0.9600
C2—H2A	0.9600	C41—H41C	0.9600
C2—H2B	0.9600	C37—H37A	0.9600
C2—H2C	0.9600	C37—H37B	0.9600
C10—C11	1.404 (2)	C37—H37C	0.9600
C10—C15	1.404 (2)	C38—H38A	0.9600
C10—N3	1.4355 (17)	C38—H38B	0.9600
C11—C12	1.393 (2)	C38—H38C	0.9600
C11—C19	1.514 (2)	C34—H34A	0.9300
C12—C13	1.370 (3)		
			
N1—B1—N3	84.01 (10)	H20B—C20—H20C	109.5
N1—B1—Br1	116.64 (10)	C19—C21—H21A	109.5
N3—B1—Br1	113.07 (10)	C19—C21—H21B	109.5
N1—B1—Br2	114.31 (10)	H21A—C21—H21B	109.5
N3—B1—Br2	118.89 (10)	C19—C21—H21C	109.5
Br1—B1—Br2	108.54 (7)	H21A—C21—H21C	109.5
C35—C30—C31	121.57 (13)	H21B—C21—H21C	109.5
C35—C30—N1	117.20 (12)	C14—C15—C10	117.62 (15)
C31—C30—N1	121.22 (12)	C14—C15—C16	118.35 (15)
C32—C31—C30	117.41 (14)	C10—C15—C16	124.02 (14)
C32—C31—C39	118.27 (14)	C15—C16—C18	111.76 (16)
C30—C31—C39	124.31 (13)	C15—C16—C17	110.57 (16)
C33—C32—C31	121.75 (14)	C18—C16—C17	109.95 (17)
C33—C32—H32A	119.1	C15—C16—H16A	108.2
C31—C32—H32A	119.1	C18—C16—H16A	108.2
C34—C33—C32	119.98 (14)	C17—C16—H16A	108.2
C34—C33—H33A	120.0	C16—C18—H18A	109.5
C32—C33—H33A	120.0	C16—C18—H18B	109.5
C34—C35—C30	118.28 (14)	H18A—C18—H18B	109.5
C34—C35—C36	119.60 (13)	C16—C18—H18C	109.5
C30—C35—C36	122.11 (13)	H18A—C18—H18C	109.5
C38—C36—C35	111.41 (15)	H18B—C18—H18C	109.5
C38—C36—C37	111.05 (19)	C16—C17—H17A	109.5
C35—C36—C37	110.85 (15)	C16—C17—H17B	109.5
C38—C36—H36A	107.8	H17A—C17—H17B	109.5
C35—C36—H36A	107.8	C16—C17—H17C	109.5
C37—C36—H36A	107.8	H17A—C17—H17C	109.5
C31—C39—C41	111.78 (15)	H17B—C17—H17C	109.5
C31—C39—C40	111.36 (15)	C1—N1—C30	127.43 (12)
C41—C39—C40	109.41 (15)	C1—N1—B1	87.79 (10)
C31—C39—H39A	108.1	C30—N1—B1	133.42 (11)
C41—C39—H39A	108.1	C1—N2—C3	120.85 (13)
C40—C39—H39A	108.1	C1—N2—C2	121.96 (13)
N2—C1—N3	130.55 (13)	C3—N2—C2	117.19 (13)
N2—C1—N1	129.12 (13)	C1—N3—C10	129.57 (12)
N3—C1—N1	100.31 (11)	C1—N3—B1	87.86 (10)
N2—C1—B1	177.85 (13)	C10—N3—B1	135.94 (11)
N3—C1—B1	50.24 (8)	C12—C13—C14	120.09 (15)
N1—C1—B1	50.08 (8)	C12—C13—H13A	120.0
N2—C3—H3A	109.5	C14—C13—H13A	120.0
N2—C3—H3B	109.5	C13—C14—C15	121.46 (16)
H3A—C3—H3B	109.5	C13—C14—H14A	119.3
N2—C3—H3C	109.5	C15—C14—H14A	119.3
H3A—C3—H3C	109.5	C39—C40—H40A	109.5
H3B—C3—H3C	109.5	C39—C40—H40B	109.5
N2—C2—H2A	109.5	H40A—C40—H40B	109.5
N2—C2—H2B	109.5	C39—C40—H40C	109.5
H2A—C2—H2B	109.5	H40A—C40—H40C	109.5
N2—C2—H2C	109.5	H40B—C40—H40C	109.5
H2A—C2—H2C	109.5	C39—C41—H41A	109.5
H2B—C2—H2C	109.5	C39—C41—H41B	109.5
C11—C10—C15	121.56 (13)	H41A—C41—H41B	109.5
C11—C10—N3	116.76 (13)	C39—C41—H41C	109.5
C15—C10—N3	121.67 (13)	H41A—C41—H41C	109.5
C12—C11—C10	117.82 (15)	H41B—C41—H41C	109.5
C12—C11—C19	119.66 (14)	C36—C37—H37A	109.5
C10—C11—C19	122.51 (13)	C36—C37—H37B	109.5
C13—C12—C11	121.40 (16)	H37A—C37—H37B	109.5
C13—C12—H12A	119.3	C36—C37—H37C	109.5
C11—C12—H12A	119.3	H37A—C37—H37C	109.5
C11—C19—C21	110.66 (17)	H37B—C37—H37C	109.5
C11—C19—C20	112.06 (17)	C36—C38—H38A	109.5
C21—C19—C20	110.67 (19)	C36—C38—H38B	109.5
C11—C19—H19A	107.8	H38A—C38—H38B	109.5
C21—C19—H19A	107.8	C36—C38—H38C	109.5
C20—C19—H19A	107.8	H38A—C38—H38C	109.5
C19—C20—H20A	109.5	H38B—C38—H38C	109.5
C19—C20—H20B	109.5	C33—C34—C35	120.97 (14)
H20A—C20—H20B	109.5	C33—C34—H34A	119.5
C19—C20—H20C	109.5	C35—C34—H34A	119.5
H20A—C20—H20C	109.5		
			
C35—C30—C31—C32	−2.1 (2)	N2—C1—N1—C30	−35.8 (2)
N1—C30—C31—C32	179.12 (13)	N3—C1—N1—C30	145.49 (13)
C35—C30—C31—C39	176.93 (14)	B1—C1—N1—C30	146.78 (17)
N1—C30—C31—C39	−1.8 (2)	N2—C1—N1—B1	177.41 (16)
C30—C31—C32—C33	1.8 (2)	N3—C1—N1—B1	−1.29 (12)
C39—C31—C32—C33	−177.33 (15)	C35—C30—N1—C1	133.90 (14)
C31—C32—C33—C34	−0.3 (3)	C31—C30—N1—C1	−47.3 (2)
C31—C30—C35—C34	1.0 (2)	C35—C30—N1—B1	−95.00 (18)
N1—C30—C35—C34	179.75 (12)	C31—C30—N1—B1	83.80 (19)
C31—C30—C35—C36	−179.47 (14)	N3—B1—N1—C1	1.11 (10)
N1—C30—C35—C36	−0.7 (2)	Br1—B1—N1—C1	−111.73 (11)
C34—C35—C36—C38	57.9 (2)	Br2—B1—N1—C1	120.17 (11)
C30—C35—C36—C38	−121.71 (19)	N3—B1—N1—C30	−142.10 (14)
C34—C35—C36—C37	−66.3 (2)	Br1—B1—N1—C30	105.05 (15)
C30—C35—C36—C37	114.09 (18)	Br2—B1—N1—C30	−23.04 (19)
C32—C31—C39—C41	−65.9 (2)	C1—B1—N1—C30	−143.21 (18)
C30—C31—C39—C41	115.01 (17)	N3—C1—N2—C3	160.85 (16)
C32—C31—C39—C40	56.8 (2)	N1—C1—N2—C3	−17.5 (2)
C30—C31—C39—C40	−122.28 (17)	N3—C1—N2—C2	−19.8 (3)
N1—B1—C1—N3	178.34 (15)	N1—C1—N2—C2	161.86 (16)
Br1—B1—C1—N3	−87.84 (12)	N2—C1—N3—C10	−23.0 (3)
Br2—B1—C1—N3	92.96 (13)	N1—C1—N3—C10	155.72 (13)
N3—B1—C1—N1	−178.34 (15)	B1—C1—N3—C10	154.42 (18)
Br1—B1—C1—N1	93.81 (13)	N2—C1—N3—B1	−177.39 (16)
Br2—B1—C1—N1	−85.39 (13)	N1—C1—N3—B1	1.29 (12)
C15—C10—C11—C12	1.4 (2)	C11—C10—N3—C1	123.75 (16)
N3—C10—C11—C12	−179.42 (13)	C15—C10—N3—C1	−57.0 (2)
C15—C10—C11—C19	−178.23 (15)	C11—C10—N3—B1	−94.59 (19)
N3—C10—C11—C19	1.0 (2)	C15—C10—N3—B1	84.6 (2)
C10—C11—C12—C13	0.4 (2)	N1—B1—N3—C1	−1.12 (10)
C19—C11—C12—C13	−179.94 (16)	Br1—B1—N3—C1	115.33 (11)
C12—C11—C19—C21	−63.0 (2)	Br2—B1—N3—C1	−115.63 (11)
C10—C11—C19—C21	116.55 (19)	N1—B1—N3—C10	−152.53 (15)
C12—C11—C19—C20	61.0 (2)	Br1—B1—N3—C10	−36.1 (2)
C10—C11—C19—C20	−119.40 (19)	Br2—B1—N3—C10	92.96 (17)
C11—C10—C15—C14	−2.5 (2)	C1—B1—N3—C10	−151.4 (2)
N3—C10—C15—C14	178.37 (13)	C11—C12—C13—C14	−1.1 (3)
C11—C10—C15—C16	176.50 (15)	C12—C13—C14—C15	−0.1 (3)
N3—C10—C15—C16	−2.7 (2)	C10—C15—C14—C13	1.8 (2)
C14—C15—C16—C18	−66.2 (2)	C16—C15—C14—C13	−177.22 (16)
C10—C15—C16—C18	114.88 (19)	C32—C33—C34—C35	−1.0 (2)
C14—C15—C16—C17	56.7 (2)	C30—C35—C34—C33	0.6 (2)
C10—C15—C16—C17	−122.29 (17)	C36—C35—C34—C33	−178.94 (15)
